# Possible Role of Bent Structure of Methylated Lithocholic Acid on Artificial and Plasma Membranes

**DOI:** 10.3390/membranes12100997

**Published:** 2022-10-14

**Authors:** Tomoyuki Iwasaki, Nobuyuki Endo, Yuta Nakayama, Toshiyuki Kamei, Toshinori Shimanouchi, Hidemi Nakamura, Keita Hayashi

**Affiliations:** 1 Division of Medical Research Support of the Advanced Research Support Center, Ehime University, Shitsukawa, Toon 791-0295, Ehime, Japan; 2 Department of Chemical Engineering, National Institute of Technology, Nara College, 22 Yata-cho, Yamatokoriyama 639-1080, Nara, Japan; 3 Graduate School of Environmental and Life Science, Okayama University, 3-1-1 Tsushimanaka, Okayama 700-8530, Okayama, Japan

**Keywords:** steroid molecule, lipid membrane, membrane property

## Abstract

Bile acids form micelles that are essential for the absorption of dietary lipids. However, excessive bile acid micelles can disrupt the plasma membrane by removing phospholipids, resulting in cell death. We hypothesized that the bent geometrical structure of the steroid scaffold of bile acids decreases the lipid order (similar to unsaturated phospholipids with *cis* double bonds), disrupting the plasma membrane. Here, lithocholic acid (LCA), a bile acid, was methylated to prevent micellization. Methylated lithocholic acid (Me-LCA) was mixed with a thin phase-separated lipid bilayer comprising 1,2-dioleoyl-*sn*-glycero-3-phosphocholine (DOPC), 1,2-dipalmitoyl-*sn*-glycero-3-phosphocholine (DPPC), and cholesterol (Chol). Me-LCA was not localized in the DPPC-rich rigid phase but localized in the DOPC-rich fluid phase, and excess Me-LCA did not affect the phase separation. Me-LCA is distributed in the plasma and organelle membranes. However, Me-LCA with bent structure did not affect the membrane properties, membrane fluidity, and hydrophobicity of liposomes composed of DOPC, DPPC, and Chol and also did not affect the proliferation of cells.

## 1. Introduction

The plasma membrane is composed of several lipid types that play key biological roles. The localization of these lipids in the plasma membrane is regulated; for example, the localization of phosphatidylserine (PS) in the inner leaflet is regulated by flippases [1,2,3]. When cells undergo apoptosis, PS translocates from the inner to the outer leaflet [4]. PS functions as an “eat me” signal, causing macrophages to engulf apoptotic cells that display PS [5]. Moreover, the localization of lipids in the plasma membrane is regulated not only by proteins but also spontaneously. Cholesterol (Chol) is an essential component of the plasma membrane [6,7]. Chol plays a vital role in forming a rigid domain in the plasma membrane, the so-called raft structure [8]. Fluid and rigid domains coexist in the plasma membrane. Whereas fluid domains are composed of unsaturated phospholipids with *cis* double bonds, rigid domains are composed of saturated phospholipids. Chol accumulates spontaneously into rigid domains and helps form raft structures. These domains play an important role in regulating signal transduction through the plasma membrane; for example, signal transduction mediated by the epidermal growth factor receptor (EGFR) [9].

The mechanism of spontaneous Chol accumulation in the membranes is unclear. Regen et al. studied the interactions between phospholipids and Chol using the nearest-neighbor recognition method with derivatives of 1-palmitoyl-2-oleoyl-*sn*-glycero-3-phosphocholine (POPC), 1,2-dipalmitoyl-*sn*-glycero-3-phosphocholine (DPPC), and Chol. The nearest-neighbor interaction free energy between Chol derivative and POPC derivative is 0.0 ± 7.9 cal/mol in the liquid-ordered (L_o_) phase and +160 ± 30 cal/mol in the liquid-disordered (L_d_) phase, and that between Chol derivative and DPPC derivative is −260 ± 6.3 cal/mol in L_o_ phase and +12 ± 19 cal/mol in L_d_ phase [10]. Here, L_o_ phase is distinct from the solid-ordered L_β_ gel phase and the liquid-disordered L_α_ fluid phase, and L_d_ phase is regarded as L_α_ phase. This difference suggests that Chol preferentially interacts with saturated phospholipids over unsaturated phospholipids via *cis* double bonds. Thus, unsaturated and saturated phospholipids push and pull Chol, facilitating lipid raft formation [11].

**Figure 1:**
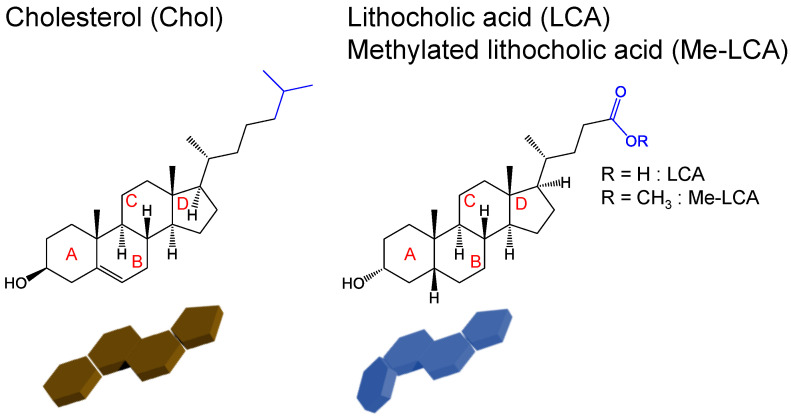
Chemical structures and simplified geometrical structures of Chol, LCA, and Me-LCA.

Although the formation of the plasma membrane is regulated by these mechanisms, other molecules can disrupt the plasma membrane. Bile acids are steroid molecules, similar to Chol, but they typically disrupt the plasma membrane [12,13]. Figure 1 shows the chemical structures and schematic images of Chol and lithocholic acid (LCA), a bile acid. Bile acids can form micelles owing to their carboxylic acid groups. The micelles suspend the dietary lipids and enhance their absorption. However, bile acids also suspend phospholipids in the plasma membrane, resulting in disruption of the plasma membrane and cell death.

Moreover, the geometrical structure of LCA differs from that of Chol because of A/B ring junctions [14]. LCA has a bent structure, although Chol has a flat structure. It has not been clear how the difference affects the localization of LCA and the contribution of raft formation such as Chol. In unsaturated acyl chains of phospholipids, *cis* double bonds decrease the lipid order [15]. Therefore, the bent structure of LCA may perturb the plasma membrane structure, resulting in cell death. However, the effects of bent structures on plasma membranes have not been thoroughly investigated.

In this study, the effects of the bent structure of LCA were investigated and compared with those of the flat structure of Chol. To evaluate the effects of the bent structure and not micellization, methylated LCA (Me-LCA) was employed (Figure 1). To evaluate the localization of Me-LCA, pyrene-conjugated LCA (Py-LCA) and (*S*)-(+)-4-(*N*,*N*-dimethylaminosulfonyl)-7-(3-aminopyrrolidin-1-yl)-2,1,3-benzoxadiazole (DBD-APy)-conjugated LCA were used as the fluorescent probes. Py-LCA was added to the thin lipid bilayers and giant unilamellar vesicles (GUVs) with L_d_ phase and L_o_ phase, and the Me-LCA phase localization was evaluated. DBD-APy-LCA was added to the cells, and the cells were observed using confocal laser fluorescence microscopy. Liposomes were used to evaluate the effect of Me-LCA on the plasma membrane. The membrane fluidity and hydrophobicity of liposomes containing Me-LCA were evaluated, and the relationship between the effects of these properties and cytotoxicity was investigated.

## 2. Materials and Methods

### 2.1. Materials

DOPC and DPPC were purchased from NOF Corporation (Tokyo, Japan), Chol from Sigma-Aldrich (St. Louis, MO, USA), and LCA from the Tokyo Chemical Industry (Tokyo, Japan). To evaluate the localization of LCA derivatives, 1,2-dioleoyl-*sn*-glycero-3-phosphoethanolamine-*N*-(lissamine rhodamine B sulfonyl) (ammonium salt) (Rho-DOPE), BODIPY-conjugated Chol (BODIPY-Chol), Py-LCA, DRAQ5, Filipin III, and DBD-APy-LCA were used as fluorescence probes. Rho-DOPE was purchased from Sigma-Aldrich (St. Louis, MO, USA), BODIPY-Chol and Filipin III from the Cayman Chemical Company (Ann Arbor, MI, USA), and DRAQ5 from BioStatus Ltd. (Loughborough, UK). Py-LCA and DBD-APy-LCA were synthesized by conjugation with 1-chloromethylpyrene and DBD-APy, respectively, purchased from Tokyo Chemical Industry (Tokyo, Japan). 4-(4,6-Dimethoxy-1,3,5-triazin-2-yl)-4-methylmorpholinium chloride *n*-hydrate (DMT-MM) purchased from FUJIFILM Wako Pure Chemical Corporation (Osaka, Japan) was conjugated with DBD-APy-LCA. The fluorescence probes used to evaluate the membrane properties of liposomes, 1,6-diphenyl-1,3,5-hexatriene (DPH), and 6-dodecanoyl-*N*,*N*-dimethyl-2-naphthylamine (Laurdan) were purchased from Sigma-Aldrich Corp. (St. Louis, MO, USA). Phosphate-buffered saline (PBS) used for MTT assay was purchased from Takara Bio Inc. (Shiga, Japan). Chol and Me-LCA were dissolved in PBS using MβCD purchased from Sigma-Aldrich Corp. (St. Louis, MO, USA). *N*,*N*-dimethylformamide (DMF), diethyl ether (DEE), hydrochloric acid solution, *n*-hexane, methanol (MeOH), sulfuric acid (H_2_SO_4_), hydrogen peroxide (H_2_O_2_), chloroform, dimethyl sulfoxide (DMSO), tetrahydrofuran (THF), and ethanol (EtOH) were purchased from FUJIFILM Wako Pure Chemical Corporation (Osaka, Japan), and 4 N HCl/dioxane from Watanabe Chemical Industries, Ltd. (Hiroshima, Japan).

### 2.2. Conjugation of Pyrene with LCA

Py-LCA was synthesized by conjugating LCA to 1-chloromethylpyrene (Figure S1). LCA (0.60 mmol), 1-chloromethylpyrene (0.60 mmol), and tetramethylammonium hydroxide pentahydrate (0.33 mmol) were added to DMF (3 mL). The mixture was then stirred for 50 min at 80 °C. The products were extracted with DEE from a diethyl ether-3.5–3.7% hydrochloric acid solution. DEE was removed using a rotary evaporator, and the products were added to *n*-hexane (100 mL) and stirred overnight. The solution was filtered to remove the precipitate, and *n*-hexane was removed using a rotary evaporator. The product was dissolved in a small amount of MeOH (~3 mL) via ultrasonication. This solution was stored overnight at −20 °C. The solution was filtered to obtain a precipitate, which was washed with cold EtOH. The purified Py-LCA was characterized by liquid-state ^1^H NMR spectroscopy at 400 MHz (JNM-ECX-400 (JEOL Co., Ltd., Tokyo, Japan)) (Figure S2) and fast atom bombardment mass spectrometry (FAB/MS) (JMS-700 (JEOL Co., Ltd., Tokyo, Japan)). ^1^H-NMR (400 MHz, CDCl_3_) δ 8.31–8.01 (m, 9H), 5.84 (s, 2H), 3.65–3.57 (m, 1H), 2.46–2.26 (m, 2H), 1.92–0.84 (m, 33H), 0.53 (s, 3H); MS (FAB) calcd for C_41_H_52_O_3_ ([M + H]^+^) 591.4, found 591.5.

### 2.3. Synthesis of Me-LCA

To methylate LCA (Figure S3), LCA (1 mmol) was dissolved in MeOH (4.5 mL), and 4 N HCl/dioxane (0.5 mL) was mixed. The solution was stirred overnight, followed by adding excess water. The product was precipitated and collected through filtration. This precipitate was then vacuum dried and characterized by liquid-state ^1^H NMR spectroscopy at 400 MHz (JNM-ECX-400 (JEOL Co., Ltd., Tokyo, Japan)) (Figure S4) and FAB/MS (JMS-700 (JEOL Co., Ltd., Tokyo, Japan)). ^1^H-NMR (400 MHz, CDCl_3_) δ 3.66 (s, 3H), 3.66–3.58 (m, 1H), 2.39–2.17 (m, 2H), 1.98–0.86 (m, 33H), 0.63 (s, 3H); MS (FAB) calcd for C_25_H_43_O_3_ ([M + H]^+^) 391.3, found 391.2.

### 2.4. Observation of Thin Lipid Bilayer Using Fluorescence Microscopy

Thin lipid bilayers were prepared according to a method from a previous paper [16]. Glass coverslips (18 mm square) were cleaned with piranha solution (95 wt% H_2_SO_4_:30 wt% H_2_O_2_ [4:1, *v*/*v*]) for 1 h at room temperature and washed extensively with water. The coverslips were stored in a desiccator and dried. Dried coverslips were placed in an electric muffle furnace (TMF-5000, Tokyo Rikakikai Co., Ltd., Tokyo, Japan). The temperature was increased from 25 to 400 °C for 1 h (6.3 °C/min), and the coverslips were baked at 400 °C for 3 h. DOPC, DPPC, Chol, and Me-LCA were dissolved in chloroform (5 μL) to a concentration of 20 mg/mL. Fluorescent probe solutions of Rho-DOPE in chloroform (1 mM), BODIPY-Chol in DMSO (1 mM), and Py-LCA in THF (1 mM) were prepared. The fluorescent probes were mixed at a molar ratio of 1 mol% of fluorescent probes, indicating (Rho-DOPE or BODIPY-Chol or Py-LCA) mol/(DOPC and DPPC and Chol) mol × 100. The mixed solution was evaporated using a rotary evaporator, and the thin lipid bilayer was dried overnight in a desiccator. Water (150 μL) was added to the thin bilayer, and the suspension was subjected to five freeze-thaw cycles. The suspension was dropped on a coverslip and dried for 2 d in a humidified atmosphere at 60 °C. The coverslip was dropped on 20 vol% MeOH, and a thin lipid bilayer was observed using fluorescence microscopy IX-51-11FL/PH-S (Olympus Corp., Tokyo, Japan).

### 2.5. Preparation and Observation of GUVs

The GUVs were prepared by gentle hydration using glucose [17]. DOPC, DPPC, Chol, and Me-LCA were dissolved in chloroform (50 μL), and the total lipid concentration was adjusted to 1 mM. The fluorescence probe solutions, Rho-DOPE in chloroform (1 mM), BODIPY-Chol in DMSO (1 mM), and Py-LCA in THF (1 mM), were added at a molar ratio of 1 mol%. Glucose dissolved in MeOH (20 mM, 25 μL) was added. The solution was added to a glass test tube, and the solvent was removed using a rotary evaporator. The thin lipid bilayer was dried in a desiccator and then hydrated with water (500 μL) and incubated for 24 h at 60 °C. The suspension was dropped on a depression slide and observed using a fluorescence microscope (IX-51-11FL/PH-S; Olympus Corp., Tokyo, Japan).

### 2.6. Cells and Cell Culture

Hep G2 and HeLa cells (JCRB1054 and JCRB9004; JCRB Cell Bank, Osaka, Japan) were cultured in Dulbecco’s modified Eagle’s medium (DMEM) containing 10% fetal bovine serum (FBS) in a humidified atmosphere of 5.0% CO_2_ at 37 °C.

### 2.7. Synthesis of DBD-APy-LCA

Conjugation of (*S*)-(+)-4-(*N*,*N*-dimethylaminosulfonyl)-7-(3-aminopyrrolidin-1-yl)-2,1,3-benzoxadiazole (DBD-APy) with LCA was performed, as shown in Figure S5. DBD-APy (0.030 mmol), LCA (0.030 mmol), triethylamine (0.063 mmol), and diphenylphosphoryl azide (DPPA) (0.033 mmol) were dissolved in THF (1 mL). The solution was then stirred in the dark for 24 h. The organic solvent was removed using a rotary evaporator. The product was dried for 2–3 days in a desiccator. MeOH (3 mL) was then added to it and stirred overnight. The product was then centrifuged, and MeOH (1 mL) was added and centrifuged. The collected product was dried for 2–3 days in a desiccator. The synthesized DBD-APy-LCA was characterized using liquid-state ^1^H NMR spectroscopy at 400 MHz (JNM-ECX-400 [JEOL Co., Ltd., Tokyo, Japan]) (Figure S6) and FAB/MS (JMS-700 [JEOL Co., Ltd., Tokyo, Japan]). ^1^H-NMR (400 MHz, DMSO-*d*_6_) δ 8.18 (d, *J* = 6.2 Hz, 1H), 7.82 (d, *J* = 8.2 Hz, 1H), 6.19 (d, *J* = 8.5 Hz, 1H), 4.46 (s, 1H), 4.41–4.36 (m, 1H), 2.66 (s, 6H), 2.28–2.20 (m, 2H), 2.13–0.80 (m, 33H), 0.47 (s, 3H); MS (FAB) calcd for C_36_H_56_N_5_O_5_S ([M + H]^+^) 670.4, found 670.4.

### 2.8. Observation of Cells Treated with DBD-APy-LCA Using a Confocal Laser Fluorescence Microscopy

Hep G2 and HeLa cells (1.0 × 10^5^ cells/mL) were cultured in DMEM containing 10% FBS. After incubation for 24 h in a humidified atmosphere of 5% CO_2_ at 37 °C, the culture medium was replaced, and then, 2 mL of 10% FBS containing DMEM and 5 μL of 7.6 mM Filipin III in DMSO was added, followed by incubation for 1 h. Then, 10 µL of 20 µM DBD-APy-LCA in DMSO and 2 µL of 5 mM DRAQ5 were added to the cells and incubated for 0.5 h. These cells were observed via confocal laser fluorescence microscopy (A1R+; Nikon, Tokyo, Japan) using the following filters: *λ*_ex_ = 352 nm and *λ*_em_ = 461 nm for Filipin III; *λ*_ex_ = 504 nm and *λ*_em_ = 511 nm for DBD-Apy-LCA, and *λ*_ex_ = 590–595 nm and *λ*_em_ = 655–660 nm for DRAQ5. Not Py-LCA, but DBD-Apy-LCA was employed because the spectrum of Py-LCA is similar to that of Filipin III, and it is difficult to separate the fluorescence between Py-LCA and Filipin III. These cells were cultured for 24 h, followed by adding 1.7 mM BODIPY-Chol in DMSO. These cells were observed after incubation for 0.5 h via confocal laser fluorescence microscopy using the following filters: *λ*_ex_ = 504 nm and *λ*_em_ = 511 nm for BODIPY-Chol.

### 2.9. Preparation of Liposomes

Model plasma membranes and liposomes were prepared using a previously described film hydration method [18]. The liposome components (DOPC, DPPC, Chol, and Me-LCA) were dissolved in chloroform. Chloroform was removed using a rotary evaporator. After drying overnight under vacuum, the residual thin membrane was hydrated with water. The suspension was subjected to five freeze–thaw cycles and extruded to produce 100 nm diameter particles.

### 2.10. DPH Fluorescence Polarization Measurement

DPH was dissolved in THF, with its concentration adjusted to 2.15 mM. The lipid concentration of the liposome suspension was adjusted to 0.1 mM using water, and 1.85 μL of the DPH solution was mixed with 10 mL liposome suspensions. The final concentrations of DPH and lipids were 0.4 μM and 0.1 mM (DPH:lipids = 1:250 [mol/mol]), and the volume ratio of THF in which DPH was dissolved was less than 1 vol%. Fluorescence intensity measurements and anisotropy evaluations were performed using an RF-5300PC fluorometer (Shimadzu Corp., Kyoto, Japan) equipped with polarizing plates. DPH was excited at 360 nm, and the fluorescence intensity at 430 nm was measured. When fluorescence intensity was measured, the temperature was maintained at 10–50 °C. The fluorescence polarization *p* value was calculated as follows:

(1) $$P=\frac{I_{vv}-\frac{I_{hv}}{I_{hh}}\times I_{vh}}{I_{vv}+\frac{I_{hv}}{I_{hh}}\times I_{vh}}$$

where *I* is the fluorescence intensity, and subscripts *v* and *h* indicate the orientation (vertical and horizontal) of the excitation and analyzer polarizers, respectively [19].

### 2.11. Evaluation of Hydrophobicity Using Fluorescence Probes

The fluorescence spectra of some fluorescence probes are solvent-dependent, and this phenomenon was applied to evaluate the hydrophobicity of the vesicles. Laurdan is an environmentally responsive fluorescent probe, and its hydrophobicity was evaluated at the boundary surface between the hydrophilic and hydrophobic regions of vesicles (Figure S7) [20]. Laurdan was dissolved in EtOH, and the concentration was adjusted to 1 mM. The lipid concentration of the liposome suspensions was adjusted to 0.1 mM using water, and 10 μL of Laurdan solution was mixed with 10 mL of liposome suspensions. Final concentration of Laurdan and lipids was 1 μM and 0.1 mM (Laurdan:lipids = 1:100 [mol/mol]). The volume ratio of EtOH in which Laurdan was dissolved was less than 1 vol%. The fluorescence spectra of Laurdan were measured using an RF-5300PC fluorometer (Shimadzu Corp., Kyoto, Japan). Hydrophobicity was evaluated using the GP_340_ values [21,22,23]. The values are calculated as follows:

(2) $${\mathrm{GP}}_{340}=\frac{I_{440}-I_{490}}{I_{440}+I_{490}}$$

where *I*_440_ and *I*_490_ are the fluorescence intensities at 440 and 490 nm, respectively, when Laurdan is excited at 340 nm.

Pyrene is also an environmentally responsive fluorescent probe [24,25], and pyrene is used to evaluate the hydrophobicity of micelles [26]. Moreover, the conjugation of dicarboxylic acids (succinic acid, suberic acid, and dodecanedioic acid) can evaluate gradual hydrophobicity in the hydrophobic region (Figure S7) [27]. The pyrene-succinic acid conjugate (Py-C_3_-COOH), pyrene-suberic acid conjugate (Py-C_7_-COOH), and pyrene-dodecanedioic acid conjugate (Py-C_11_-COOH) were dissolved in THF, and their concentrations were adjusted to 10 mM. The solution was then mixed with chloroform to dissolve the lipids. Liposomes were prepared and adjusted to 100 nm, as described above. The ratio of the pyrene-dicarboxylic acid conjugates to lipids was 1:100 [mol/mol]. The prepared liposome suspension was diluted, and the final concentrations of the pyrene−dicarboxylic acid conjugate and lipids were 1 μM and 0.1 mM. Pyrene-dicarboxylic acid conjugates were excited at 336 nm. The fluorescence spectra were measured from 350 to 600 nm at 10–50 °C using an RF-5300PC fluorometer (Shimadzu Corp., Kyoto, Japan). Hydrophobicity was evaluated based on the fluorescence intensity of peaks I (377 ± 2 nm, *I*_I_) and III (387 ± 2 nm, *I*_III_). The ratio of *I*_I_ to *I*_III_ (*I*_I_/*I*_III_) is related to the relative permittivity of aliphatic monoalcohols. The correlation with the relative permittivity was fitted using the following equation:

(3) IIIIII=α−β×γεr

where *α*, *β*, and *γ* are constants, *ε*_r_ corresponds to the relative permittivity, and each equation of Py-C3-COOH, Py-C7-COOH, and Py-C11-COOH has already been reported [27]. The relative permittivity of the liposomes was estimated from the *I*_I_/*I*_III_ values based on the correlation equations.

### 2.12. MTT Assay

The MTT assay was performed using MTT Cell Count Kit (Nacalai Tesque, Kyoto, Japan). Hep G2 cells and HeLa cells were seeded on 96-well culture plates (100 μL, 2.0 × 10^5^ cells/mL) and cultured in DMEM (with 10% FBS) in an incubator. After 24 h of growth, the following formulations (3 μL) were added to the cells: Chol and Me-LCA diluted in PBS and treated with 200 mM MβCD (Sigma-Aldrich, St. Louis, MO, USA). After incubation for 24 h, 10 µL MTT solution was added to the cells. After incubation for 3 h, 100 μL of solubilization solution was added, and the formazan precipitate was dissolved by pipetting. The absorbance of each well was measured at 570 and 650 nm using a microplate spectrophotometer (Flex Station; Molecular Devices, San Jose, CA, USA). The cell viability was calculated using the following equation:

(4) $$\mathrm{Relative}\;\mathrm{cell}\;\mathrm{viability}=\frac{A_{570}-A_{650}}{A_{570}{}_{\left(0\right)}-A_{650}{}_{\left(0\right)}}$$

where *A*_570_ and *A*_650_ are the absorbance values at 570 and 650 nm, respectively, when cells were treated with the sample. *A*_570(0)_ and *A*_650(0)_ are the absorbance values when the cells were treated with 200 mM MβCD in PBS. The absorbance of formazan was maximum at a wavelength of 570 nm, and absorbance at 650 nm (reference wavelength) was measured to reduce the effect of cell debris.

### 2.13. Statistical Analysis

All experiments were performed three times, and the results were expressed as the mean ± standard deviation.

## 3. Results

### 3.1. Localization of Me-LCA in Phospholipid Bilayers and Plasma Membranes

**Figure 2:**
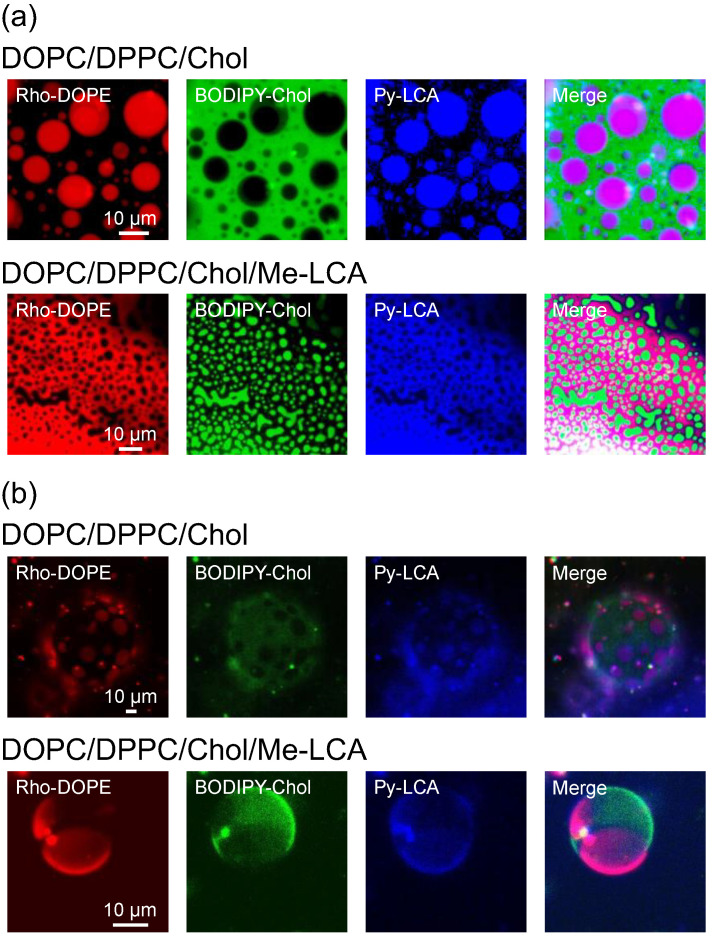
Observation of Rho-DOPE (red), BODIPY-Chol (green), and Py-LCA (blue) in (**a**) lipid thin bilayers and (**b**) GUVs using fluorescence microscopy.

It was unclear if the bile acid derivative tested herein was localized in plasma membranes. Then, we used an artificial plasma membrane composed of phospholipids and Chol as a control system relative to the plasma membrane. The fluorescence probes, Rho-DOPE, BODIPY-Chol, and Py-LCA were thereby mixed in thin lipid bilayers, and the bilayers were observed by fluorescence microscopy (Figure 2a). When a thin lipid bilayer composed of DOPC/DPPC/Chol (1:1:1, mol/mol) was observed, the DOPC-rich L_d_ phase and DPPC- and Chol-rich L_o_ phases coexisted on the liposomal membrane at approximately 25 °C [28,29,30]. While the Rho-DOPE was localized in the L_d_ phase, the BODIPY-Chol was localized in the L_o_ phase. The fluorescence of Rho-DOPE and BODIPY-Chol was observed in different parts of the thin lipid bilayer because of the phase separation. Py-LCA fluorescence is observed in the L_d_ phase. This result suggests that Me-LCA is localized in the L_d_ phase even though it is a steroid molecule similar to Chol, which is localized in the L_o_ phase. Excessive secretion of bile acids may lead to their accumulation in the plasma membrane. To investigate the behavior of Me-LCA in plasma membranes containing a large number of Me-LCA molecules, DOPC/DPPC/Chol/Me-LCA (1:1:1:1, mol/mol) thin lipid bilayer was also employed. The fluorescence of Rho-DOPE and Py-LCA was observed in the same part of the thin lipid bilayer. These fluorescence probes and BODIPY-Chol were observed in different parts of the thin lipid bilayer. Thus, the thin lipid bilayer was heterogeneous; despite the presence of a large number of Me-LCA molecules (25 mol%), Py-LCA was localized in the L_d_ phase. The area of the L_d_ phase was increased because the number of components in the L_d_ phase (i.e., Me-LCA) increased.

The Py-LCA localization was studied using GUVs (Figure 2b), and the result using GUVs is similar to that obtained using thin lipid bilayers. Me-LCA did not affect the phase separation, and Py-LCA localized in the L_d_ phase of GUVs was composed of DOPC, DPPC, Chol, and Me-LCA.

**Figure 3:**
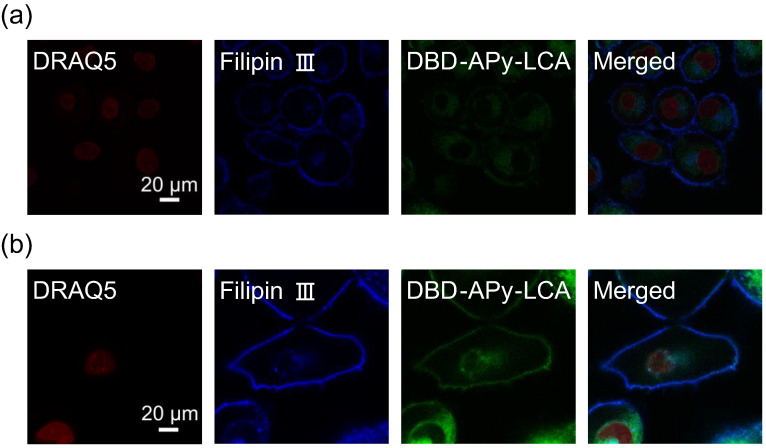
Confocal laser fluorescence microscopy images of (**a**) Hep G2 and (**b**) HeLa cells treated with DRAQ5 (red), Filipin III (blue), and DBD-APy-LCA (green).

Next, Hep G2 and HeLa cells were used for the target system. To investigate the localization of LCA, LCA was labeled with the chromophore DBD-APy to synthesize DBD-APu-LCA for investigating the cellular localization of Me-LCA. DBD-APy-LCA was added to both Hep G2 and HeLa cells, which were then observed by confocal laser fluorescence microscopy. These cells were also treated with DRAQ5 as a fluorescent DNA stain and Filipin III as a fluorescent Chol stain. Figure 3 shows confocal laser microscopy images of Hep G2 and HeLa cells treated with these fluorescence probes. DBD-APy-LCA fluorescence was observed in the plasma membrane, similar to that of Filipin III. DBD-APy-LCA was detected in the cytoplasm. This result shows that some DBD-APy-LCA molecules were localized in the plasma membrane, whereas other molecules were localized in the cytoplasm. This result suggests that Me-LCA accumulates in the plasma and organelle membranes.

### 3.2. Evaluation of Effects of Me-LCA on Membrane Properties and Comparison with Chol

It was unclear if the localization of Me-LCA associated with its staining the plasma (and organelle) membranes had an impact on the membrane property of plasma membranes of cells from the observation using fluorescence and confocal laser microscopy in the last section. Accordingly, in this section, we examined the influence of Me-LCA on membrane properties such as (i) DPH fluorescence polarization, (ii) hydrophobicity at the boundary surface between the headgroup and acyl chain region, and (iii) hydrophobicity in the hydrophobic region.

**Figure 4:**
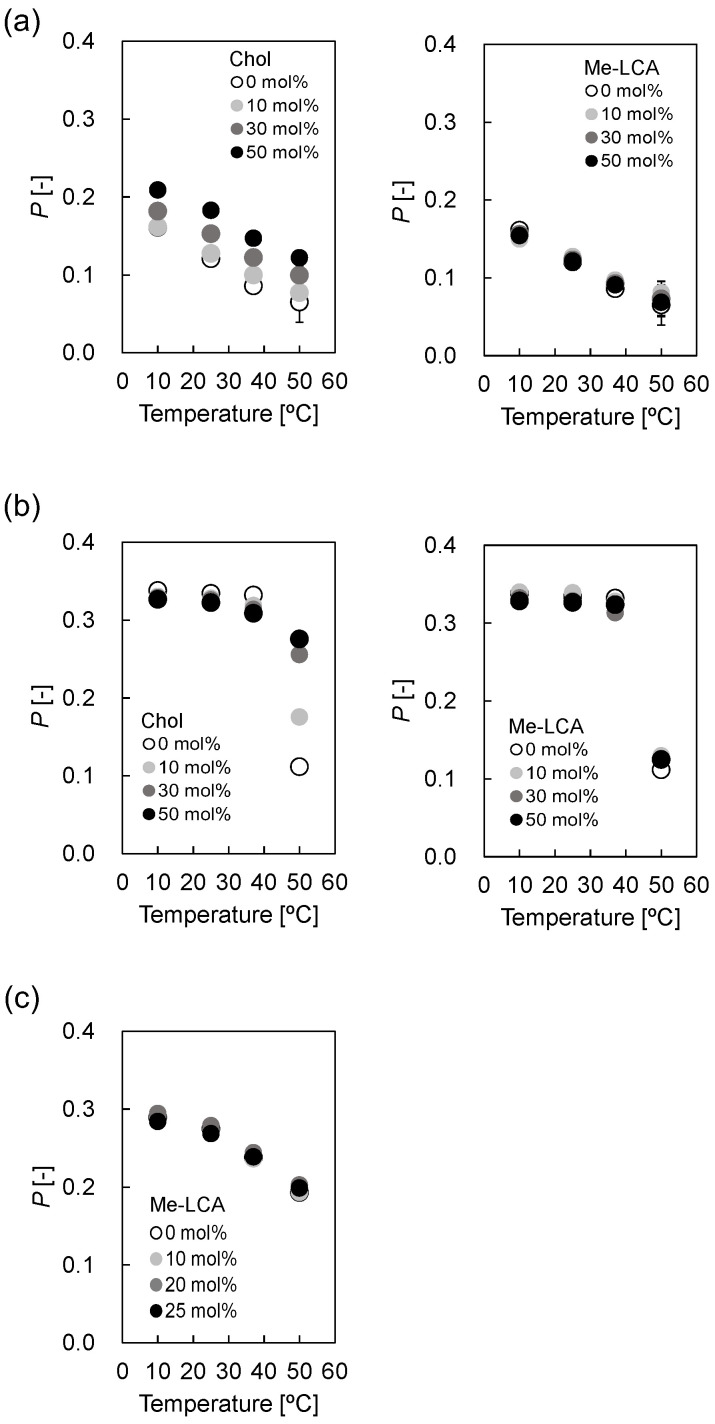
*p* values of (**a**) DOPC liposome, (**b**) DPPC liposome, and (**c**) DOPC/DPPC/Chol (1:1:1, mol/mol) liposome with Me-LCA.

For (i), DPH fluorescence polarization measurements were used to evaluate liposomal microviscosity [19]. Figure 4a shows the fluorescence polarization *p* values of DOPC liposomes containing Chol and Me-LCA. The *p* values of DOPC liposomes increased with increasing Chol molar ratio at 10 °C. An increase in temperature reduced the *p* values of DOPC liposomes, regardless of the Chol molar ratio, but the *p* values of DOPC liposomes increased with increasing Chol molar ratio, independent of temperature. Furthermore, the *p* values remained nearly constant regardless of the Me-LCA molar ratio at 10–50 °C. Figure 4b shows the *p* values of DPPC liposomes. The *p* values of DPPC liposomes were similar regardless of whether steroid molecules were present or not at ≤37 °C. The *p* values were different at 50 °C for DPPC liposomes. Although the *p* value of DPPC liposomes without Chol decreased remarkably with a change in temperature from 37 °C to 50 °C, this decrease was suppressed by Chol. The smallest decrease was observed for 50 mol% Chol. The *p* values of DPPC liposomes with 0–50 mol% Me-LCA were also measured, but no difference was observed, regardless of the Me-LCA molar ratio, unlike Chol at 10–50 °C. The *p* value of DPPC liposomes with 0 mol% Me-LCA decreased remarkably with a change in temperature from 37 °C to 50 °C, and the *p* values of DPPC liposomes with 10–50 mol% Me-LCA changed similarly. This decrease seems to correlate with the gel (P_β_’) to liquid crystalline (L_α_) phase transition of DPPC lipid bilayers whose phase transition temperature is 41.3 °C [31]. Figure 4c shows the *p* values of DOPC/DPPC/Chol (1:1:1, mol/mol) liposomes with 0–25 mol% Me-LCA. The *p* values were similar regardless of the Me-LCA molar ratio and temperature. The *p* values decreased with increasing temperature, similar to DOPC liposomes with Me-LCA.

**Figure 5:**
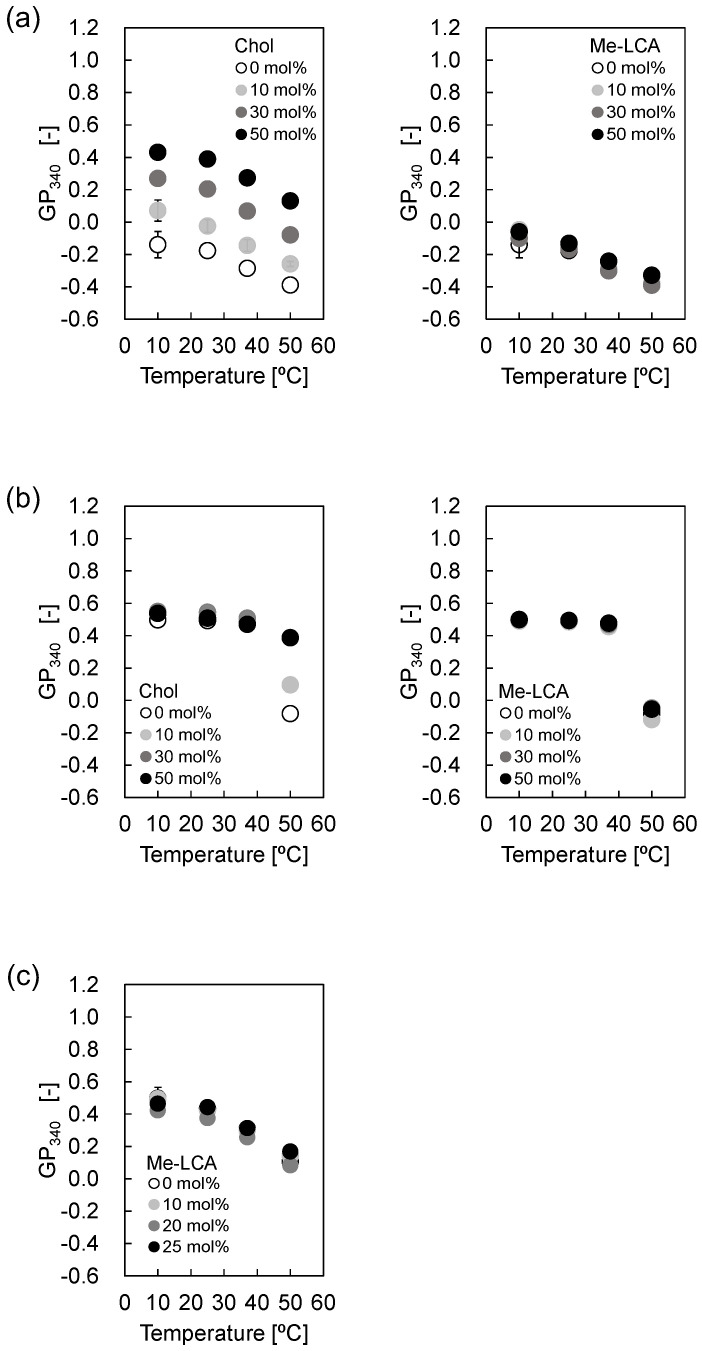
GP_340_ values of (**a**) DOPC liposome, (**b**) DPPC liposome, and (**c**) DOPC/DPPC/Chol (1:1:1, mol/mol) liposome with Me-LCA.

For (ii), the hydrophobicity at the boundary surface between the headgroup and acyl chain region was evaluated using a Laurdan fluorescence probe. Laurdan was used to evaluate hydrophobicity based on the GP_340_ values calculated from the fluorescence spectrum [21,22,23]. Figure 5a shows the GP_340_ values of DOPC liposomes with Chol or Me-LCA. Chol increased GP_340_ values, whereas Me-LCA did not affect GP_340_ values in a temperature-dependent manner. Figure 5b shows GP_340_ values of DPPC liposomes. Variations in GP_340_ values were not observed regardless of the presence or absence of steroid molecules at <40 °C. While Chol increased the GP_340_ values at 50 °C, Me-LCA did not affect the GP_340_ values. Figure 5c shows the GP_340_ values of DOPC/DPPC/Chol (1:1:1, mol/mol) liposomes; Me-LCA did not affect the GP_340_ values at 10–50 °C.

**Figure 6:**
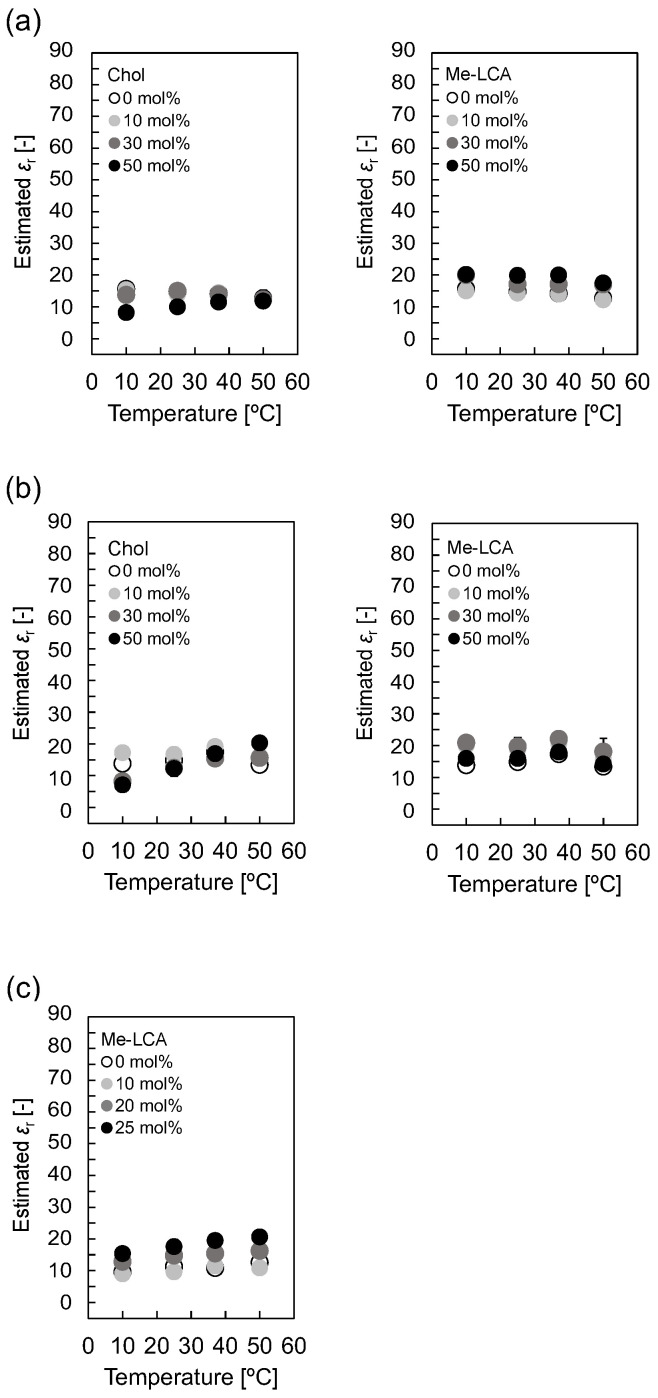
Estimated *ε*_r_ values using Py-C_7_-COOH of (**a**) DOPC liposome, (**b**) DPPC liposome, and (**c**) DOPC/DPPC/Chol (1:1:1, mol/mol) liposome with Me-LCA.

For (iii), the hydrophobicity in the hydrophobic region was evaluated using fluorescence probes and pyrene-dicarboxylic acid conjugates (Py-C_3_-COOH, Py-C_7_-COOH, and Py-C_11_-COOH). Pyrene was used to evaluate hydrophobicity based on values of *I*_I_/*I*_III_, which is the ratio of the intensities of peak I (377 ± 2 nm, *I*_I_) and peak III (387 ± 2 nm, *I*_III_) of the pyrene fluorescence spectrum. *I*_I_/*I*_III_ values were used to estimate the relative permittivity, and Py-C_3_-COOH, Py-C_7_-COOH, and Py-C_11_-COOH were evaluated in the shallow, middle, and deep regions [27]. Figure 6 shows the estimated *ε*_r_ of liposomes using Py-C_7_-COOH. The estimated *ε*_r_ of the DOPC liposomes with 0–50 mol% Chol was 8–16 at 10–50 °C, and a remarkable difference was hardly observed, regardless of Chol. In addition, Me-LCA did not affect the estimated *ε*_r_ of DOPC liposomes. Moreover, the estimated *ε*_r_ values of DPPC liposomes and DOPC/DPPC/Chol (1:1:1, mol/mol) liposomes were similar, regardless of the molar ratio of Chol to Me-LCA. Figures S8 and S9 show the estimated *ε*_r_ values of liposomes using Py-C_3_-COOH and Py-C_11_-COOH. These results also showed that Me-LCA did not significantly affect the estimated *ε*_r_ of the liposomes.

From the results, Me-LCA was unlikely to alter the membrane properties tested herein.

### 3.3. Cytotoxicity of Me-LCA

**Figure 7:**
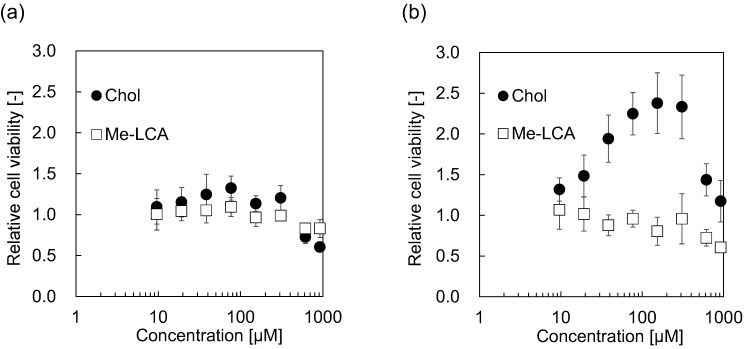
Cell viability of (**a**) Hep G2 and (**b**) HeLa cells treated with Chol or Me-LCA.

To confirm the impact of Me-LCA on cells or their plasma membranes, cell viability was also examined. Hep G2 and HeLa cells that are well-known were treated with Chol or Me-LCA, and the viability of these cells was evaluated using the MTT assay (Figure 7). When Hep G2 cells were treated with Chol and Me-LCA at concentrations <100 μM, Chol and Me-LCA did not affect relative cell viability. High concentrations of Chol and Me-LCA decreased relative cell viability. In contrast, the relative viability of HeLa cells showed different changes after treatment with Chol or Me-LCA. When HeLa cells were treated with Chol at concentrations <150 μM, relative cell viability increased. However, the relative cell viability decreased from 2.4 to 1.2 at higher concentrations. Me-LCA did not increase the relative cell viability. Changes in the relative viability of HeLa cells treated with Me-LCA were similar to those of Hep G2 cells. Treatment with 300 μM Me-LCA did not affect the relative viability of HeLa cells, whereas a high concentration of Me-LCA decreased the relative cell viability.

## 4. Discussion

We hypothesized the bent structure of Me-LCA might contribute to the order structure of artificial and plasma membranes. As a matter of fact, the impact of Me-LCA was quite different from that of Chol. That is, Me-LCA did not alter the membrane properties of DOPC, DPPC, and DOPC/DPPC/Chol (1:1:1) (Figure 4, Figure 5 and Figure 6). Furthermore, the additive effect of Me-LCA different from Chol did not alter the cell viability of Hep G2 and HeLa cells (Figure 7). These results could be comprehensive from the data concerning artificial membranes. Herein, we discuss why Me-LCA did not alter the viability of cells in terms of the membrane property.

**Figure 8:**
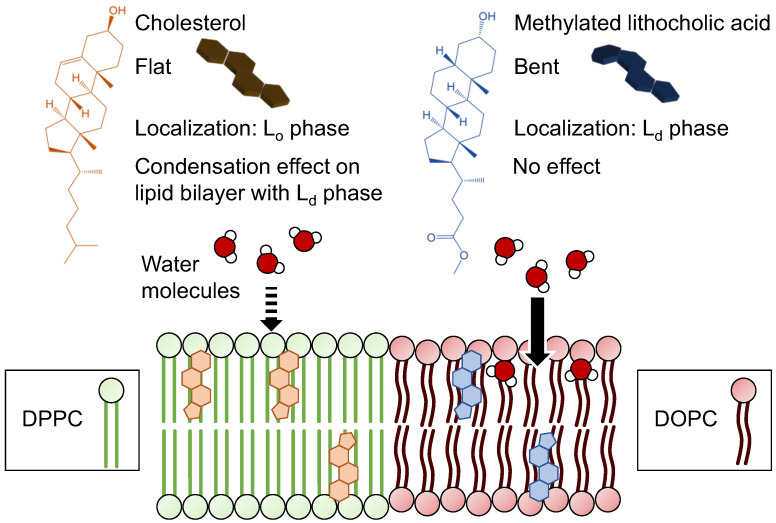
A schematic image of differences in localization and an effect on membrane property between Chol and Me-LCA.

The cytotoxicity of bile acids is well documented. A detergent model of cell death induced by bile acids posits that bile acids remove phospholipids from the plasma membrane [12,13]. In this study, to investigate the possibility that the bent structure of bile acids contributes to cell death, localization and effects of Me-LCA were evaluated (Figure 8). For example, hydrophilic bile acid, sodium cholate is at the head group area [32]. However, hydrophobic Me-LCA has only one hydrophilic hydroxyl group. Thus, Me-LCA is in the lipid bilayer such as Chol, whose 3-OH faces an aqueous phase [33]. Me-LCA, which cannot form micelles, was used as a model bile acid. The localization of Me-LCA was initially investigated because the effects of Me-LCA on the lipid bilayer may depend on the phase state. To evaluate the effects of Me-LCA, Chol, which has a flat structure, was used. Previous studies report a relationship between the localization of Chol and the effects of Chol on the lipid bilayer. The condensation effect of Chol can produce many close hydrophobic contacts with neighboring acyl chains of phospholipids, resulting in tight packing [34,35]. Moreover, Chol becomes a repulsive phospholipid as the proportion of *cis* double bonds increases [36]. Thus, Chol pulls saturated phospholipids and pushes away unsaturated phospholipids with *cis* double bonds, inducing a phase separation [11]. However, this condensation effect is not unique to Chol. Other steroid molecules such as 7-dehydrocholesterol, campesterol, β-sitosterol, ergosterol, brassicasterol, and stigmasterol also increase lipid chain order [37].

In contrast, Me-LCA, a steroid molecule derivative, did not increase lipid chain order. As shown in Figure 4, membrane fluidity was evaluated using DPH. Me-LCA did not affect the *p* value, whereas Chol decreased the *p* value. The difference between these steroid molecules containing Chol and Me-LCA is their geometrical structures. We assumed that the flat structure of Chol is suitable for interaction with the neighboring acyl chains of phospholipids, but the bent structure of Me-LCA is further away from the hydrophobic contacts. This difference was also related to its localization in the lipid bilayer. While BODIPY-Chol was localized in the L_o_ phase, Py-LCA was localized in the L_d_ phase (Figure 2). This result suggests that Chol prefers saturated phospholipids to bile acids and that it pushes bile acids to the L_d_ phase. Therefore, such an action might contribute to the localization of the Me-LCA to the L_d_ phase of plasma membranes.

When cells were treated with DBD-APy-LCA, fluorescence was observed in both the plasma membrane and the cytosol (Figure 3). DBD-APy-LCA is taken up by the cytosol via endocytosis and/or bile acid receptors. Filipin III binds to Chol in the plasma membrane and fluoresces in the plasma membrane. However, when cells were treated with BODIPY-Chol, fluorescence was observed in the cytosol (Figure S10). Chol is delivered by apolipoproteins in the body, and its distribution is regulated [38]. In contrast, the distribution of dissolved Chol molecules in DMSO is not regulated compared to that in the body. Dissolved Chol molecules are distributed in the cytosol through the plasma membrane. Therefore, both Me-LCA and Chol-dissolved steroid molecules affect the properties of the plasma and organelle membranes.

Besides, Me-LCA neither condenses the phospholipids nor perturbs the lipid bilayer (Figure 4, Figure 5 and Figure 6). When the lipid bilayer is perturbed by other molecules, such as amphiphilic peptides, the *p* value decreases [39]. Such perturbation promotes the penetration of water molecules into the lipid bilayer [40], which suggests that the GP_340_ value decreases and the estimated *ε*_r_ value increases. However, Me-LCA did not affect *p* value, GP_340_ value, and estimated *ε*_r_. These results suggest that Me-LCA with a bent structure hardly perturbs the lipid bilayer, relating to cytotoxicity. Me-LCA did not affect the proliferation of Hep G2 and HeLa cells. The cytotoxicity of deoxycholic acid (DC), chenodeoxycholic acid (CDCA), glycochenodeoxycholic acid (GCDCA), and LCA on Hep G2 cells was evaluated. DC, CDCA, and GCDCA at 750 μM were found to lyse Hep G2 cells after 4 h of incubation [41]. As shown in Figure 7, the relative cell viability of Hep G2 and HeLa cells treated with Me-LCA at >750 μM was higher than that of Hep G2 cells treated with DC, CDCA, and GCDCA at 750 μM. Evaluation of cytotoxicity suggested that bile acid micelles mainly contributed to cell death by removing phospholipids constituting the plasma membrane. However, LCA did not affect the proliferation of Hep G2 cells [41]. One possible reason for this is the deprotonation of the carboxy group. DCA, CDCA, and GCDCA are expected to deprotonate more easily than LCA. DCA, CDCA, and GCDCA also easily form micelles. Thus, micelle-formable cholic acid derivatives, including DCA, CDCA, and GCDCA, inhibited the proliferation of Hep G2 cells as compared to LCA.

From the discussion mentioned above, Me-LCA cannot replace Chol. Chol is a component of the raft structure that plays an important role in regulating the behavior of membrane proteins [8]. When HeLa cells were treated with Chol, their proliferation was improved (Figure 7). This phenomenon has been reported in human prostate cancer cells (PC-3 cells) [42]. EGFR is a membrane protein that promotes cell proliferation. The EGFR substrate 15-related protein (EPS15R) regulates the internalization of EGFR [43], resulting in the inhibition of proliferation. Chol affects EGFR internalization. Adipocyte plasma membrane-associated protein (APMAP) is a transmembrane protein that accumulates in the choline-induced lipid rafts. APMAP interacts with EPS15R. The interaction with EPS15R inhibited EGFR internalization and promoted cell proliferation. Meanwhile, Me-LCA is pushed out to the L_d_ phase due to its bent structure so that it cannot be localized in the raft region (Chol with a flat structure can be stably localized in the raft region). Therefore, Me-LCA cannot trigger the proliferation mechanism occurring at the raft region.

## 5. Conclusions

A methylated bile acid derivative, Me-LCA, was used to investigate cytotoxicity induced by bile acids. Me-LCA was localized in the L_d_ phase and did not perturb the lipid bilayer. Me-LCA did not affect cell proliferation, although we hypothesized that the bent geometrical structure of the steroid scaffold of bile acids contributes to inducing cell death. Moreover, the study compared the effect of Me-LCA with that of Chol, a steroid molecule similar to bile acids. Chol orders the lipid arrays; however, Me-LCA neither perturbs nor orders the membrane. Chol could increase the HeLa cell proliferation, but Me-LCA could not. These results suggest that Me-LCA is not an alternative to Chol, which exhibits a condensation effect and is a component of the raft structure. Me-LCA did not affect the membrane properties to induce cell death.

## Data Availability

The data generated during the study are available from the corresponding author, Keita Hayashi, upon request.

## Supplementary Materials

The following supporting information can be downloaded at: https://www.mdpi.com/article/10.3390/membranes12100997/s1, Figure S1: Scheme of the synthesis of Py-LCA. Figure S2: NMR spectrum of Py-LCA. Figure S3: Scheme of the synthesis of Me-LCA. Figure S4: NMR spectrum of Me-LCA. Figure S5: Scheme of the synthesis of DBD-APy-LCA. Figure S6: NMR spectrum of DBD-APy-LCA. Figure S7: Localization of fluorescence probes in liposome. Figure S8: Estimated *ε*_r_ values using Py-C_3_-COOH of (a) DOPC liposome, (b) DPPC liposome, and (c) DOPC/DPPC/Chol (1:1:1, mol/mol) liposome with Me-LCA. Figure S9: Estimated εr values using Py-C11-COOH of (a) DOPC liposome, (b) DPPC liposome, and (c) DOPC/DPPC/Chol (1:1:1, mol/mol) liposome with Me-LCA. Figure S10: Confocal laser fluorescence microscopy images of (a) Hep G2 and (b) HeLa cells treated with DRAQ5 (red) and BODIPY-Chol (green).
