# Supplementary material for: Possible Role of Bent Structure of Methylated Lithocholic Acid on Artificial and Plasma Membranes
Source: Membranes (Basel). 2022 Oct 14;12(10):997. doi: 10.3390/membranes12100997 (PMC9610195; doi:10.3390/membranes12100997)
Supplement: Supplementary file 1 [file membranes-12-00997-s001.zip › membranes-1922309-supplementary.pdf]

Supporting Information

SUPPLEMENTARY DATA

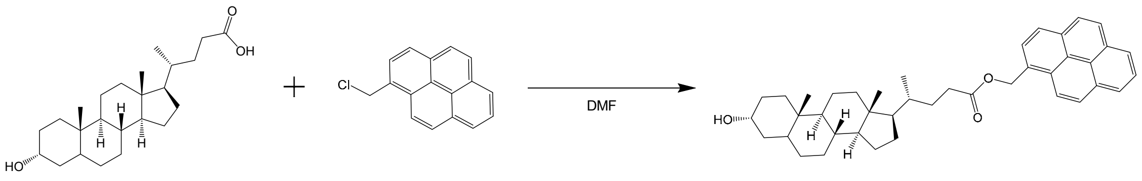

Figure. S1. Scheme of the synthesis of Py-LCA.

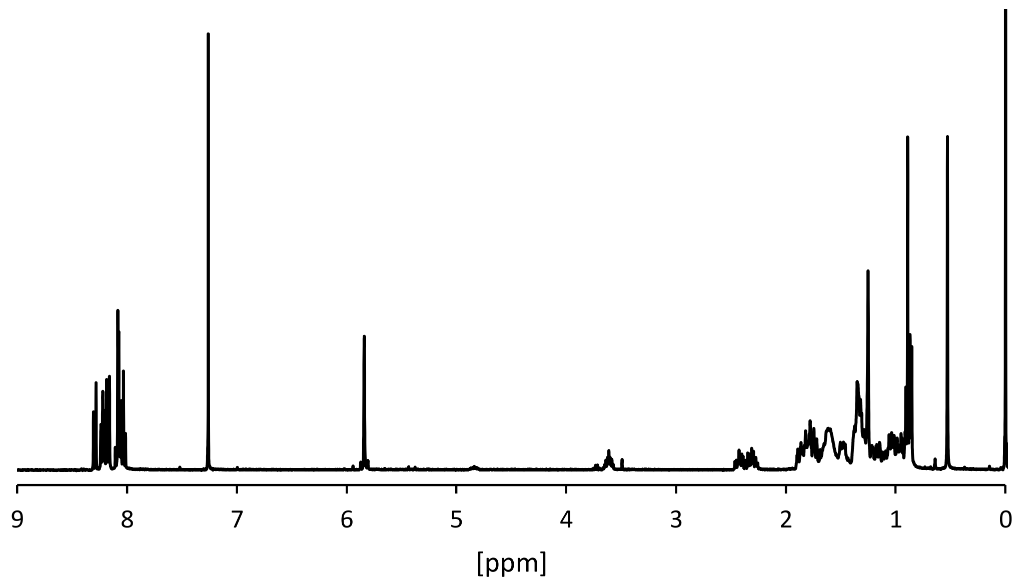

Figure. S2. NMR spectrum of Py-LCA.

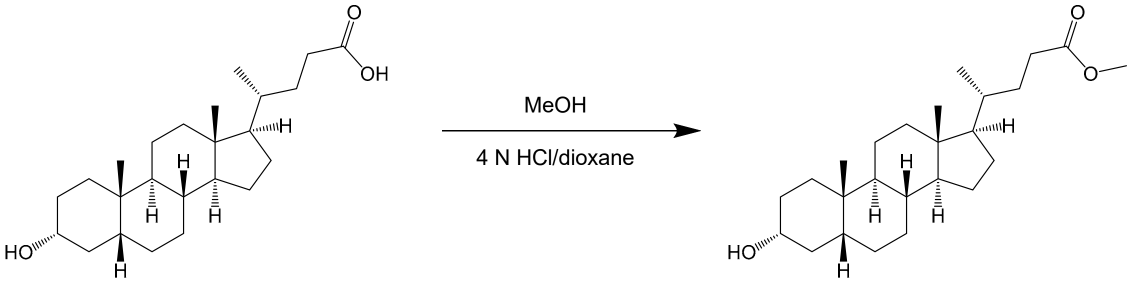

Figure. S3. Scheme of the synthesis of Me-LCA.

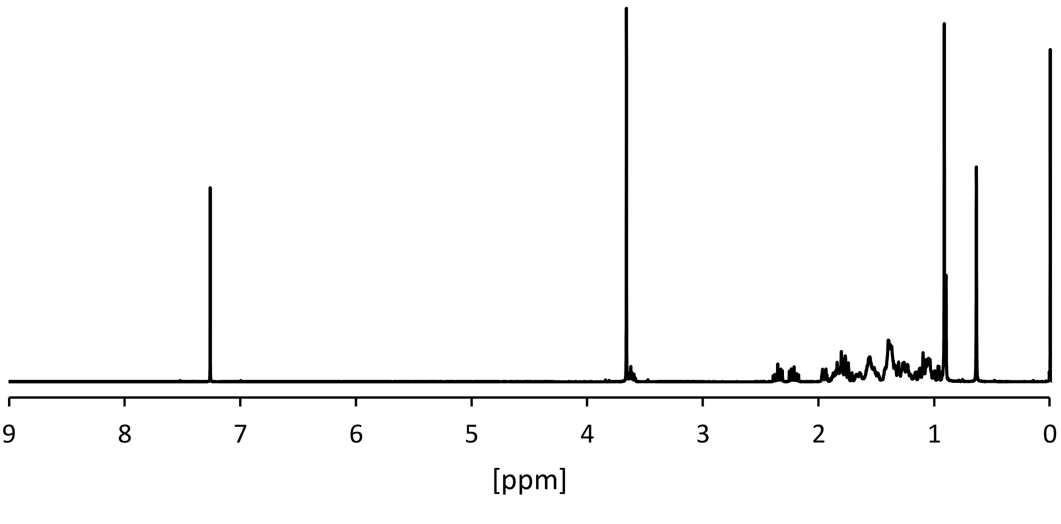

Figure. S4. NMR spectrum of Me-LCA.

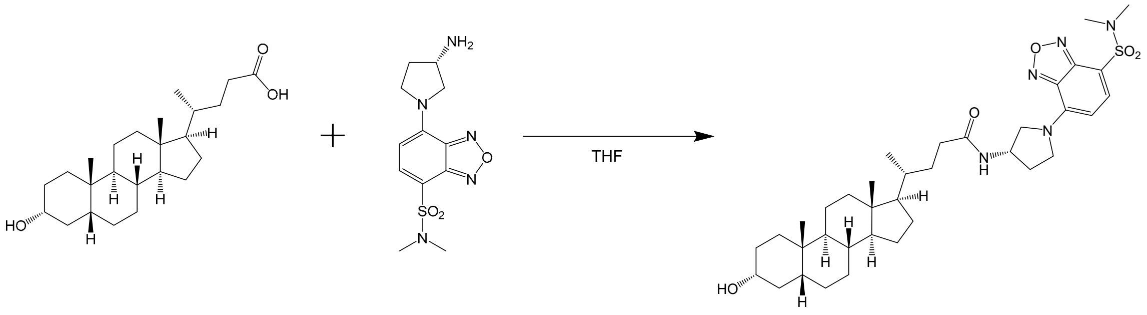

Figure. S5. Scheme of the synthesis of DBD-APy-LCA.

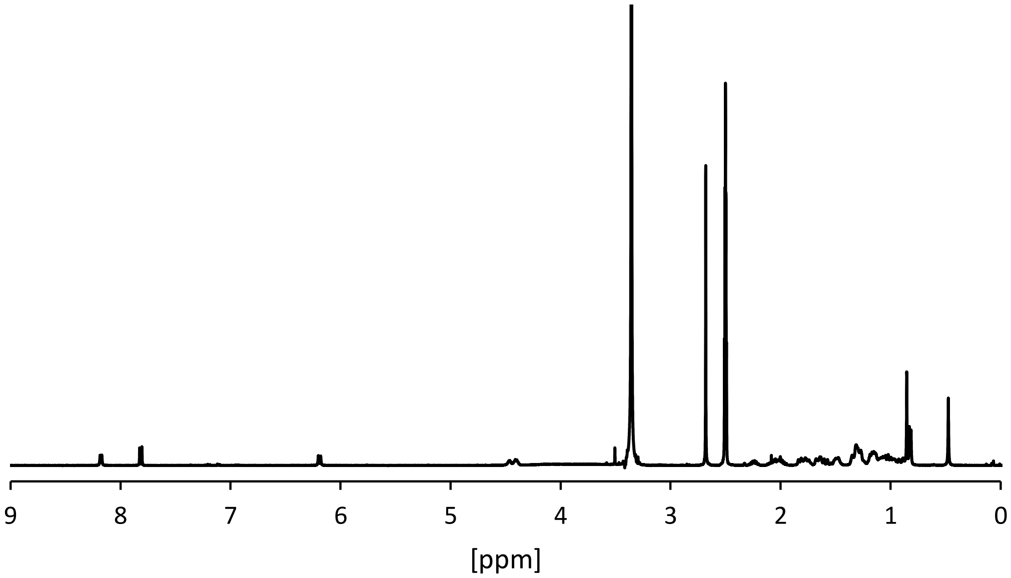

Figure. S6. NMR spectrum of DBD-APy-LCA.

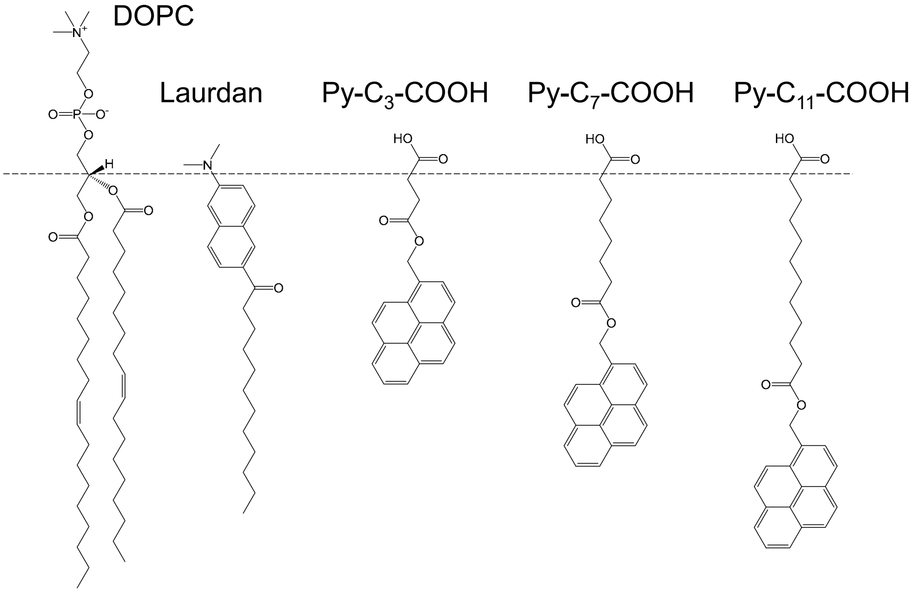

Figure. S7. Localization of fluorescence probes in liposome.

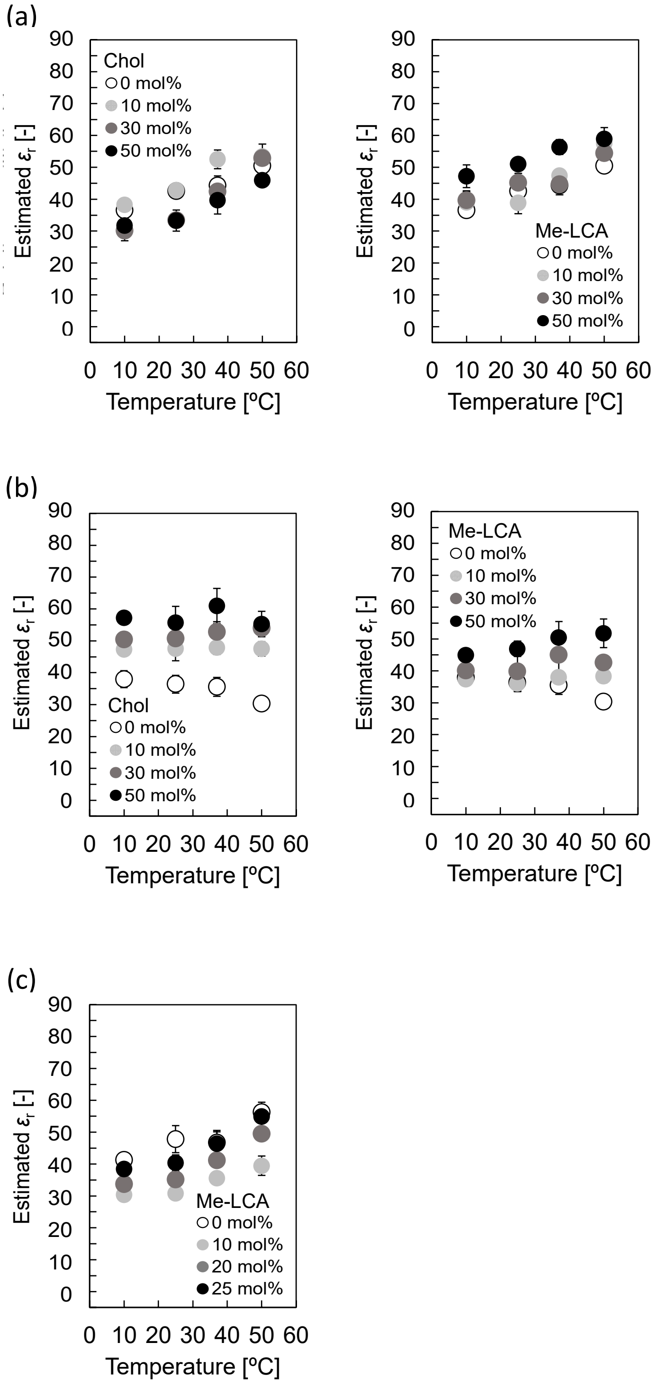

Figure. S8. Estimated ε_r values using Py-C_3-COOH of (a) DOPC liposome, (b) DPPC liposome, and (c) DOPC/DPPC/Chol (1:1:1, mol/mol) liposome with Me-LCA.

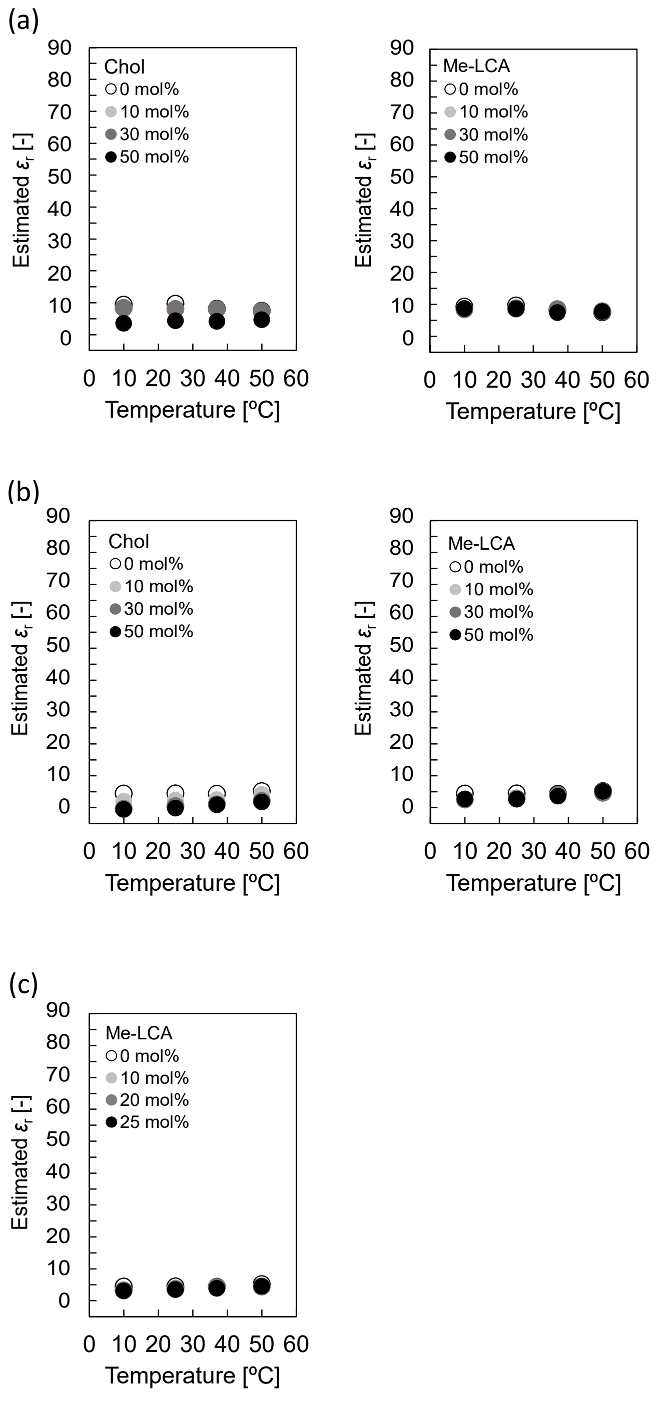

Figure. S9. Estimated ε_r values using Py-C_{11}-COOH of (a) DOPC liposome, (b) DPPC liposome, and (c) DOPC/DPPC/Chol (1:1:1, mol/mol) liposome with Me-LCA.

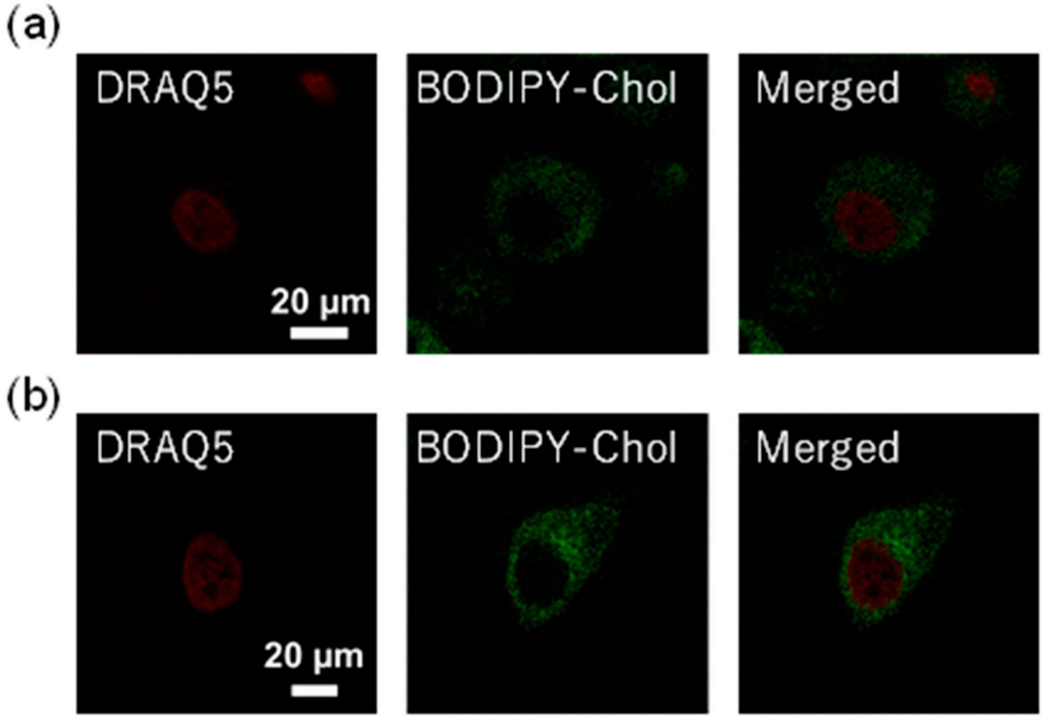

Figure. S10. Confocal laser fluorescence microscopy images of (a) Hep G2 and (b) HeLa cells treated with DRAQ5 (red) and BODIPY-Chol (green).
